# Draft Genome Sequence of *Serratia proteamaculans* MFPA44A14-05, a Model Organism for the Study of Meat and Seafood Spoilage

**DOI:** 10.1128/genomeA.00491-17

**Published:** 2017-06-08

**Authors:** Lysiane Fougy, Gwendoline Coeuret, Marie-Christine Champomier-Vergès, Stéphane Chaillou

**Affiliations:** a MICALIS Institute, INRA, AgroParisTech, Université Paris-Saclay, Domaine de Vilvert, Jouy-en-Josas, France; b Aérial, Parc d’Innovation, Illkirch, France

## Abstract

In this study, we present a draft genome sequence of *Serratia proteamaculans* MFPA44A14-05. This strain was isolated from a spoiled organic modified-atmosphere-packed beef carpaccio. The draft genome sequence will contribute to the understanding of the role of the *S. proteamaculans* species in meat and seafood spoilage.

## GENOME ANNOUNCEMENT

Members of the *Serratia* genus have a highly ubiquitous nature. Like most *Enterobacteriaceae*, they are commonly found in the digestive tracts of animals but also thrive very competitively in water and soil environments (1). Some species, like *Serratia proteamaculans*, are also well known for contaminating and spoiling protein-rich food, like raw meat and seafood (2–4), using a strong capacity to resist CO_2_-enriched modified atmosphere (MA) (5) and to metabolize protein and amino acids for growth (6). Despite this key role in food waste, very little is known about the genomic background of *S. proteamaculans*, and the genomes of only two strains, those of the plant growth-promoting strain S4 (7) and the poplar tree root endophyte strain 568 (8), are currently available.

*S. proteamaculans* strain MFPA44A14-05 was isolated in 2009 from a highly spoiled slice of organic modified atmosphere-packed beef carpaccio (9). After 14 days of storage at 8°C, the strain was dominant and had reached a population level of 6.7 log_10_ CFU g^-1^, turning the carpaccio slices into a brown/greenish color and diffusing a strong putrid smell. We thus undertook the genome sequence of this strain in order to use it as a model to understand the role of *S. proteamaculans* in meat and seafood spoilage.

The whole-genome sequencing of *S. proteamaculans* MFPA44A14-05 (CIP 110939) was carried out by Eurofins MWG Operon laboratories (Ebersberg, Germany) using Illumina MiSeq 2 × 150-bp paired-end libraries. The 2.94 million reads were assembled *de novo* using the Velvet software (10) after choosing the best k-mer value of 73. The draft assembly resulted in 80 contigs from 1,783 to 252,892 bp (*N*_50_, 128,235 bp). The contigs were aligned against the *S. proteamaculans* strain 568 complete genome using progressiveMauve (11) to give one high-quality scaffold (5,368.81 bp; coverage, 46×), with an overall G+C content of 54.85%. Annotation performed with the MicroScope platform (12) detected 5,075 coding sequences (CDSs), 29 pseudogenes, 4 rRNAs, and 76 tRNAs. The MFPA44A14-05 strain has been deposited in the CIP Culture Collection under the reference CIP 110939.

The high proteolytic capacity of *S. proteamaculans* MFPA44A14-05 is confirmed by the detection of at least 5 extracellular proteases or proteinases, including a serralysin-like proteinase (13), a subtilisin-like protease, and an overall set of 18 peptidases. Similarly, the number of genes involved in peptide and amino acid metabolism (*n* = 594) largely exceeds the number of genes involved in carbohydrate metabolism (*n* = 473). This COG group is the biggest in proportion (11% of the whole genome). We also noticed that the *S. proteamaculans* MFPA44A14-05 genome contains biogenic amine production activity arising from amino acid catabolism, which involves two genes encoding a lysine decarboxylase to produce cadaverine (*cadA* and *ldcC* genes) and all genes of the polyamine superpathway II (*speA* to* speF*) to produce agmatine, putrescine, and spermidine from arginine, ornithine, and *S*-adenosylmethionine.

### Accession number(s).

This whole-genome shotgun project has been deposited in ENA BioProject number PRJEB20089 and assembly under the accession numbers FWWG01000001 to FWWG01000080. The version described in this paper is the first version.

## References

[1] Petersen LM, Tisa LS 2013 Friend or foe? A review of the mechanisms that drive *Serratia* towards diverse lifestyles. Can J Microbiol 59:627–640. doi:10.1139/cjm-2013-0343. 24011346

[2] Säde E, Murros A, Björkroth J 2013 Predominant enterobacteria on modified-atmosphere packaged meat and poultry. Food Microbiol 34:252–258. doi:10.1016/j.fm.2012.10.007. 23541191

[3] Fougy L, Desmonts MH, Coeuret G, Fassel C, Hamon E, Hézard B, Champomier-Vergès MC, Chaillou S 2016 Reducing salt in raw pork sausages increases spoilage and correlates with reduced bacterial diversity. Appl Environ Microbiol 82:3928–3939. doi:10.1128/AEM.00323-16. 27107120 PMC4907177

[4] Macé S, Joffraud JJ, Cardinal M, Malcheva M, Cornet J, Lalanne V, Chevalier F, Sérot T, Pilet MF, Dousset X 2013 Evaluation of the spoilage potential of bacteria isolated from spoiled raw salmon (*Salmo salar*) fillets stored under modified atmosphere packaging. Int J Food Microbiol 160:227–238. doi:10.1016/j.ijfoodmicro.2012.10.013. 23290229

[5] Schuerger AC, Ulrich R, Berry BJ, Nicholson WL 2013 Growth of *Serratia liquefaciens* under 7 mbar, 0°C, and CO_2_-enriched anoxic atmospheres. Astrobiology 13:115–131. doi:10.1089/ast.2011.0811. 23289858 PMC3582281

[6] Borch E, Kant-Muermans ML, Blixt Y 1996 Bacterial spoilage of meat and cured meat products. Int J Food Microbiol 33:103–120. doi:10.1016/0168-1605(96)01135-X. 8913812

[7] Neupane S, Goodwin LA, Högberg N, Kyrpides NC, Alström S, Bruce D, Quintana B, Munk C, Daligault H, Teshima H, Davenport K, Reitenga K, Green L, Chain P, Erkkila T, Gu W, Zhang X, Xu Y, Kunde Y, Chertkov O, Han J, Han C, Detter JC, Ivanova N, Pati A, Chen A, Szeto E, Mavromatis K, Huntemann M, Nolan M, Pitluck S, Deshpande S, Markowitz V, Pagani I, Klenk HP, Woyke T, Finlay RD 2013 Non-contiguous finished genome sequence of plant-growth promoting *Serratia proteamaculans* S4. Stand Genomic Sci 8:441–449. doi:10.4056/sigs.4027757. 24501629 PMC3910699

[8] Taghavi S, Garafola C, Monchy S, Newman L, Hoffman A, Weyens N, Barac T, Vangronsveld J, van der Lelie D 2009 Genome survey and characterization of endophytic bacteria exhibiting a beneficial effect on growth and development of poplar trees. Appl Environ Microbiol 75:748–757. doi:10.1128/AEM.02239-08. 19060168 PMC2632133

[9] Lucquin I, Zagorec M, Champomier-Vergès M, Chaillou S 2012 Fingerprint of lactic acid bacteria population in beef carpaccio is influenced by storage process and seasonal changes. Food Microbiol 29:187–196. doi:10.1016/j.fm.2011.08.001. 22202872

[10] Zerbino DR 2010 Using the Velvet *de novo* assembler for short-read sequencing technologies. Curr Protoc Bioinformatics Chapter 11:Unit 11.5. doi:10.1002/0471250953.bi1105s31. PMC2952100 20836074

[11] Darling AE, Mau B, Perna NT 2010 progressiveMauve: multiple genome alignment with gene gain, loss and rearrangement. PLoS One 5:e11147. doi:10.1371/journal.pone.0011147. 20593022 PMC2892488

[12] Vallenet D, Belda E, Calteau A, Cruveiller S, Engelen S, Lajus A, Le Fèvre F, Longin C, Mornico D, Roche D, Rouy Z, Salvignol G, Scarpelli C, Thil Smith AA, Weiman M, Médigue C 2013 MicroScope—an integrated microbial resource for the curation and comparative analysis of genomic and metabolic data. Nucleic Acids Res 41:D636–D647. doi:10.1093/nar/gks1194. 23193269 PMC3531135

[13] Zhang L, Morrison AJ, Thibodeau PH 2015 Interdomain contacts and the stability of serralysin protease from *Serratia marcescens*. PLoS One 10:e0138419. doi:10.1371/journal.pone.0138419. 26378460 PMC4574703

